# Cell organelle-based analysis of cell chirality

**DOI:** 10.1080/19420889.2019.1605277

**Published:** 2019-04-24

**Authors:** Jie Fan, Haokang Zhang, Tasnif Rahman, Diana N. Stanton, Leo Q. Wan

**Affiliations:** a Department of Biomedical Engineering, Rensselaer Polytechnic Institute, Troy, NY, USA; b Center for Biotechnology & Interdisciplinary Studies, Rensselaer Polytechnic Institute, Troy, NY, USA; c Department of Biological Sciences, Rensselaer Polytechnic Institute, Troy, NY, USA; d Center for Modeling, Simulation and Imaging in Medicine, Rensselaer Polytechnic Institute, Troy, NY, USA

**Keywords:** Cell chirality, left-right asymmetry, cell polarity, endothelial cells

## Abstract

The maintenance of tight endothelial junctions requires the establishment of proper cell polarity, which includes not only the apicobasal and front-rear polarity but also the left-right (L-R) polarity. The cell possesses an intrinsic mechanism of orienting the L-R axis with respect to the other axes, following a left-hand or right-hand rule, termed cell chirality. We have previously reported that endothelial cells exhibit a clockwise or rightward bias on ring-shaped micropatterns. Now we further characterize the chirality of individual endothelial cells on micropatterns by analyzing the L-R positioning of the cell centroid relative to the nucleus-centrosome axis. Our results show that the centroids of endothelial cells preferably polarized towards the right side of the nucleus-centrosome axis. This bias is consistent with cell chirality characterized by other methods. These results suggest that the positioning of cell organelles is intrinsically L-R biased inside individual cells. This L-R bias provides an opportunity for determining cell chirality in situ, even in vivo, without the limitations of using isolated cells in in vitro engineered platforms.

Anatomical axes such as anterior-posterior (AP), dorsal-ventral (DV), and L-R axes, are often used to identify the asymmetry of organisms in vivo. Cells exhibit a notable L-R bias relative to the overall anatomical axes at certain stages of embryonic development, such as the initial stages of hindgut rotation [1] and heart looping [2]. Increasing evidence suggests that L-R symmetry breaking may be initiated by the chirality of a group of specific cells during embryogenesis [2,3]. The emergence of cell chirality (or handedness) during embryonic development may establish the L-R asymmetry of internal tissues/organs, while disturbed cell chiral biases may lead to laterality disorders, such as heterotaxy and *situs inversus totalis*. In addition, as a fundamental property of the cell, cell chirality may have significant implications in physiological processes that remain largely underexplored. Therefore, it is important to determine the chirality of cells and understand its role in development and disease so that better prevention and treatment strategies can be developed.

Traditionally, the L-R biases of the cells have been determined based on the cell polarization relative to the anatomic axes in embryonic development. However, whether and how these global (i.e., AP and DV) axes guide the L-R cell polarization is still unclear. Therefore, it is important to determine the intracellular chiral biases that are independent of the systematic factors, such as morphogen gradients, in developing embryos. One approach is to measure the chirality of the cells isolated from biological tissues using in vitro engineering tools so that the cells are not exposed to the abovementioned factors. The cell chirality in vitro, at both single cell level and multi-cellular level, was clearly demonstrated in multicellular chiral morphogenesis [4], L-R biased polarization [5–7], directional migration [8–11], and biased rotation of cell structures such as actin structure [12,13], nucleus [13], cytoplasmic flow [14], and the cell as a whole [15,16]. These studies have undoubtedly shown that chirality is a universal property of the cell. However, the inability of correlating the chiral behavior in vitro with that in vivo has greatly limited the understanding of the role of cell chirality in many developmental and physiological processes. Therefore, it is crucial to develop an intracellular marker for individual cell chirality in situ.

Towards this goal, we propose that certain organelles inside a cell may show L-R biased positioning since the cells are intrinsically chiral. Using endothelial cells, we have demonstrated that the L-R biases of the cells (or cell chirality) can be measured by the biased positioning of cell centroid (left side vs right side) relative to the front-rear polarization of the cell, which is widely characterized by the axis oriented from nucleus to centrosome, or the nucleus-centrosome axis [17–19]. Like other planar cells, endothelial cells demonstrate a clear vertical apicobasal polarization to the planar plane, restraining the front-rear and L-R axes within the plane, thus making it possible to determine the bias of each cell in the monolayer. Within a confluent human umbilical vein endothelial cells (hUVECs) monolayer, we have found the cell centroid positioning is biased to the right side of the nucleus-centrosome axis. The overall chiral biases determined with this approach is consistent with results from the multicellular patterning method [4]. We also confirmed that this new approach can be extended to study the endothelial cells in situ from the human umbilical veins, as well as those from mouse thoracic aorta and vena cava [20].

Recently we have further performed the cell-organelle based chirality analysis of hUVECs patterned on microscale ring-shape surfaces. As expected, the hUVECs showed a clockwise alignment with the cell-cell junction labeled by the ZO-1 antibody (Figure 1A). After image segmentation along cell borders, the centroid of each cell was calculated using ImageJ (Figure 1B). Similarly, the cell nuclei (labeled by DAPI) were segmented, followed by the calculation of their centroids (Figure 1C). The centrosome staining was concentrated as a single dot located next to the nucleus inside each cell. These three dots, nuclear centroid, centrosome, and cell centroid (Figure 1D), were used to characterize the cell L-R biases by judging the L-R positioning of cell centroid relative to the nucleus-centrosome vector (Figure 1E). Finally, the cells were color-coded by their biases, showing the overall distribution on the micropattern. According to this cell-organelle based analysis, significantly more hUVECs exhibited a right bias than a left bias, which was consistent with the chirality measured with the multicellular patterning method [4,20]. Here for the first time we directly demonstrate that the bias of multicellular alignment on micropatterned surfaces is consistent with the chirality of organelle positioning of the exactly same cells.

**Figure 1.**
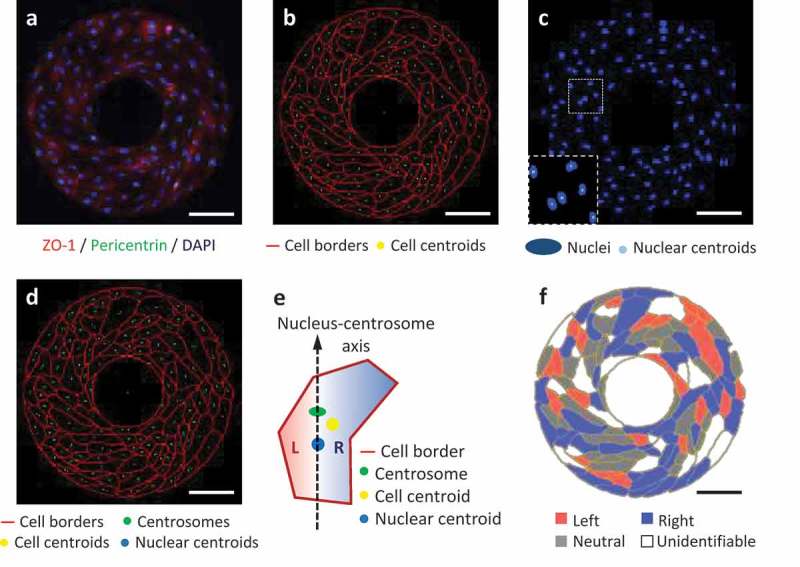
L-R biases of micropatterned hUVECs analyzed from the fluorescent images. (a) Immunofluorescence of hUVECs on a ring-shaped micropattern showing cell junctions (ZO-1, red), cell nuclei (DAPI, blue) and centrosomes (pericentrin, green). (b) Cell borders segmented from the ZO-1 channel in (a), shown with the calculated cell centroids (yellow). (c) Cell nuclei (blue) segmented from the blue channel in (a), shown with nuclear centroids (cyan). (d) Merged image for cell bias analysis, including cell borders (red), centrosomes (green), nuclear centroids (blue) and cell centroids (yellow). (e) A schematic of determination of the left (L) or right (R) cell bias according to the positioning of the cell centroid relative to the nucleus-centrosome vector. (f) Color-coded cells by their biases on the micropattern. Scale bars: 100 um.

The mechanism of this right-biased positioning of cell centroid remains unclear, but it might result from the biased migration of endothelial cells [9,10,21]. The right biased turning of the cell leading edge would continuously orient the cell shape bending towards the right side, resulting in the cell centroid falling to the right side of the front-rear axis. This speculation is supported by the previous reports that the extension of a new pseudopod in a migrating cell precedes centrosome repositioning [22]. In a cell that migrates with a rightward bias, the leading edge has a significantly higher chance of falling to the right side of the nucleus-centrosome axis. This postulation, together with the mechanism of the intricate regulation of cell organelle positioning in dynamic cell movement, needs to be closely examined with live cell imaging in the future.

There are a few limitations of this new approach. First, some cells are difficult to characterize into either left or right bias, and some endothelial cells are even categorized as left-biased. This is probably due to substantial noise associated with biophysical randomness in motion and/or stochastic gene expression of individual cells. Second, compared with the multicellular approaches such as the multicellular patterning method, the bias of positioning of the cell centroid, centrosome, and nuclear centroid may not be as robust, although it clearly has its advantages for the in vivo settings. Third, instead of the organelles used in this study, other intracellular makers may be better candidates for determining cell chirality, such as the planar cell polarity markers including aPKC, par proteins, cdc42, and Vangl2 at the cell leading edge. Fourth, this method is limited to planar cells with clear cell junctions since the calculation of cell centroid greatly relies on the clear visualization of cell borders. Therefore, it may be challenging for 3D non-planar tissue. Finally, developing an accurate, automated computational programming for the recognition of cell organelles and analysis of relative positioning could greatly advance the research of cell chirality.

## Materials and methods

### Cell patterning and chirality analysis

The human umbilical vein endothelial cells (hUVECs) were patterned and cultured on the micro ring-shape as described previously [4,20].

### Immunofluorescence and image analysis for cell biases

Micropatterned cells were fixed, permeabilized, blocked, and incubated with ZO-1 and pericentrin antibodies followed by appreciated secondary antibodies. Finally, samples were mounted in Fluoromount-G with DAPI and imaged using a fluorescence microscope (BZ-X700, Keyence). Detailed analysis for cell biases was described previously [20].
